# Incidence, trends and demographics of Staphylococcus aureus infections in Auckland, New Zealand, 2001–2011

**DOI:** 10.1186/1471-2334-13-569

**Published:** 2013-12-03

**Authors:** Deborah A Williamson, Alwin Lim, Mark G Thomas, Michael G Baker, Sally A Roberts, John D Fraser, Stephen R Ritchie

**Affiliations:** 1 Faculty of Medical and Health Sciences, University of Auckland, Auckland, New Zealand; 2 Department of Clinical Microbiology, Auckland District Health Board, Auckland, New Zealand; 3 Institute of Environmental Science and Research, Wellington, New Zealand; 4 Department of Public Health, University of Otago, Wellington, New Zealand; 5 Mailing address: Department of Molecular Medicine and Pathology, University of Auckland, Private Bag 92019, Auckland, New Zealand

**Keywords:** *Staphylococcus aureus*, Epidemiology, Healthcare-associated infection, Ethnicity, Methicillin-susceptible

## Abstract

**Background:**

New Zealand has a higher incidence of *Staphylococcus aureus* disease than other developed countries, with significant sociodemographic variation in incidence rates. In contrast to North America, the majority of disease is due to methicillin-susceptible *S. aureus* (MSSA), although relatively little is known about the comparative demographics of MSSA and methicillin-resistant *S. aureus* (MRSA) infections in New Zealand.

**Methods:**

Our objectives were to describe the trends, incidence and patient demographics of all *S. aureus* infections in patients presenting to our institution between 2001 and 2011, and compare the epidemiology of MSSA and MRSA infections. We identified all patients with *S. aureus* infections over the study period. A unique *S. aureus* infection was defined as the first positive *S. aureus* culture taken from the same patient within a thirty-day period. Standard definitions were used to classify episodes into community- or healthcare-associated *S. aureus* infection.

**Results:**

There were 16,249 *S. aureus* infections over the study period. The incidence increased significantly over the study period from 360 to 412 per 100,000 population (*P* < 0.001), largely driven by an increase in community-associated non-invasive MSSA infections. When compared with MSSA infections, patients with non-multiresistant MRSA infections were more likely to be older, have hospital-onset infections and be Māori or Pacific Peoples.

**Conclusions:**

Our work provides valuable baseline data on the epidemiology and trends of *S. aureus* infections in New Zealand. The significant increase in community-associated *S. aureus* infections is of public health importance. Future studies should investigate the reasons underlying this concerning trend.

## Background

*Staphylococcus aureus* is a major human pathogen, and infections caused by *S. aureus* result in significant morbidity and mortality [1,2]. Although most commonly associated with skin and soft tissue infections (SSTI), *S. aureus* is also responsible for a range of serious invasive infections, including osteomyelitis, necrotizing pneumonia and bacteremia [3]. In addition, *S. aureus* disease occurs commonly in both community and healthcare settings amongst a variety of demographic groups [1,4,5]. To date, many studies assessing the burden of *S. aureus* infections have focused on one particular aspect of *S. aureus* disease, such as a specific clinical syndrome (e.g. bloodstream infections or SSTI), specific patients (e.g. adults only) or a specific place of acquisition (e.g. healthcare-associated infections) [6-8]. Furthermore, as a consequence of the recent epidemic of community-associated methicillin resistant *S. aureus* (CA-MRSA) in North America, many studies have focused exclusively on describing the epidemiology and burden of MRSA infections [9,10]. As such, population-based data on the overall extent of *S. aureus* disease, regardless of clinical syndrome, healthcare exposure or antimicrobial resistance profile, are limited.

The reported incidence of *S. aureus* disease in New Zealand is higher than that from similar developed countries [11-13]. However, in contrast to several other settings, particularly North America, the vast majority of *S. aureus* disease in New Zealand is due to methicillin-susceptible *S. aureus* (MSSA) [13-15]. Distinct demographic associations have been described for *S. aureus* infections in New Zealand, including age, ethnicity and economic deprivation [11,16,17]. To date however, relatively little is known about the comparative epidemiology of infections caused by MSSA and MRSA strains in our setting. Moreover, no studies have systematically assessed longitudinal trends in the overall incidence and epidemiology of *S. aureus* infections in New Zealand. Accordingly, we sought to: (i) describe the incidence, trends and patient characteristics of *S. aureus* infections in patients presenting to our hospital over an 11-year period, and (ii) compare the demographics of infections caused by MSSA and MRSA strains.

## Methods

### Setting, patients and isolates

Auckland District Health Board (ADHB) is a tertiary-level, university-affiliated institution exclusively serving a population of approximately 500,000, within a larger metropolitan region of 1.4 million inhabitants. Auckland is the largest city in New Zealand, and has an ethnically diverse population, having the following major population groups: European (52%); Asian (29%), Pacific Peoples (11%); Māori (indigenous New Zealander, 8%) and other ethnicities (2%) [18].

All specimens that cultured *S. aureus* between January 1^st^ 2001 and December 31^st^ 2011 were identified from the laboratory database in the Department of Clinical Microbiology, Auckland City Hospital, New Zealand. In order to restrict the analysis to isolates that were likely to represent clinically relevant infections, all screening isolates (anterior nares; axillae; groin) and isolates from genital swabs, pharyngeal swabs and stool were excluded. In addition, all specimens that did not have a culture site specified were excluded. A sterile culture site was defined as one matching the US Centers for Disease Control and Prevention (CDC) surveillance systems definition [8]. A unique *S. aureus* infection was defined as the first positive *S. aureus* culture taken from the same patient within a thirty-day period. All *S. aureus* isolates were identified using standardised laboratory protocols. Antimicrobial susceptibility testing was performed by agar dilution and results were interpreted according to Clinical and Laboratory Standards Institute recommendations [19]. In keeping with previous studies, strains that were phenotypically resistant to <3 non β-lactam antimicrobials were defined as non-multidrug-resistant MRSA (nmMRSA), and strains that were resistant to ≥3 non-β-lactam antimicrobials were defined as multidrug-resistant MRSA (mMRSA) [20,21].

### Data collection

The following demographic information was extracted from the hospital administrative database about each patient who had an inpatient hospital admission associated with a positive *S. aureus* culture: age, gender, ethnicity, residence in a long term care facility, and number of previous hospitalizations in the preceding twelve months. In addition, hospital discharge diagnoses related to each hospital admission (coded using the *International Classification of Diseases, Tenth Edition,* [*ICD-10*] codes) were obtained for each patient. The NZ Deprivation index (NZDep) score was used to assign socioeconomic status to each patient. This score is an area-based measure of deprivation derived from New Zealand census data, and is based on various measures of deprivation, including: household income, household ownership, household occupancy, employment and education levels, and access to telecommunications [22]. It is expressed as a score between one and ten, a score of ten representing the most deprived neighbourhoods.

Population denominator information for the ADHB catchment region was obtained from the 2001 and 2006 New Zealand censuses, and from linear interpolation between census data for the study period [18]. For analysis, ethnicity was grouped into four major groupings: European, Māori, Pacific Peoples and Other ethnicities.

### Definitions of *Staphylococcus aureus*-related hospital admissions

Based on previously described methodology, a list of *ICD-10* codes for *S. aureus*-related clinical syndromes were developed (a detailed list of codes are shown in Additional file 1) [23-25]. These syndromes were: (i) skin and soft tissue infection (SSTI); (ii) musculoskeletal infection; (iii) respiratory infection; (iv) endovascular infection; (v) central nervous system infection, and (vi) sepsis/bacteremia. For analysis, these categories were further classified as either non-invasive infections (SSTI) or invasive infections (all other clinical syndromes). All *S. aureus* bloodstream infection were classified as invasive infections. In addition, other invasive *S. aureus* infections were defined by the isolation of *S. aureus* from a clinical specimen, plus ≥1 *ICD-10* discharge diagnoses associated with an invasive clinical syndrome (Additional file 1). For positive *S. aureus* culture results with ≥1 *ICD-10* discharge codes associated with an invasive clinical syndrome, the principal discharge code was regarded as the most likely clinical syndrome. A non-invasive infection was defined as the isolation of *S. aureus* from a non-sterile site, with a principal or secondary *ICD-10* discharge diagnosis associated with a SSTI (Additional file 1) and no *ICD-10* codes associated with an invasive clinical syndrome.

Based on definitions adapted from similar studies, [17,26] cases were described as community-associated if *S. aureus* was isolated from a patient within 48 hours of hospital admission who: (i) had no history of hospitalization or surgery in the preceding calendar year, (ii) did not reside in a long-term care facility (LTCF), and (iii) did not have any prior or current *ICD-10* discharge diagnoses relating to hemodialysis. Conversely, cases were described as healthcare-associated (HCA) *S. aureus* infection if one or more of these risk factors were documented. Healthcare-associated cases were further classified as hospital-onset (HCA-HO) or community-onset (HCA-CO) depending on whether the specimen was taken > 48 hours or ≤ 48 hours respectively, following hospital admission.

### Statistical analysis

Categorical variables were compared using the χ^2^ or Fisher’s exact test as appropriate. Non-parametric data were compared using the Mann–Whitney *U* test or Kruskal-Wallis test. Incidence rates were calculated per 100,000 population, and a Poisson log-linear regression model was used to assess trends in incidence rates using log population denominator data as the offset variable. Multivariate logistic regression analysis using a stepwise backward elimination model was used to identify factors associated with nmMRSA infections. All statistical analysis was performed using GraphPad Prism (Version 5.02) or STATA (Version 11) and a two-tailed *P* value of < 0.05 was considered significant.

### Ethics

Auckland District Health Board, New Zealand, granted institutional approval for this study.

## Results

### Patients and incidence rates

**Figure 1:**
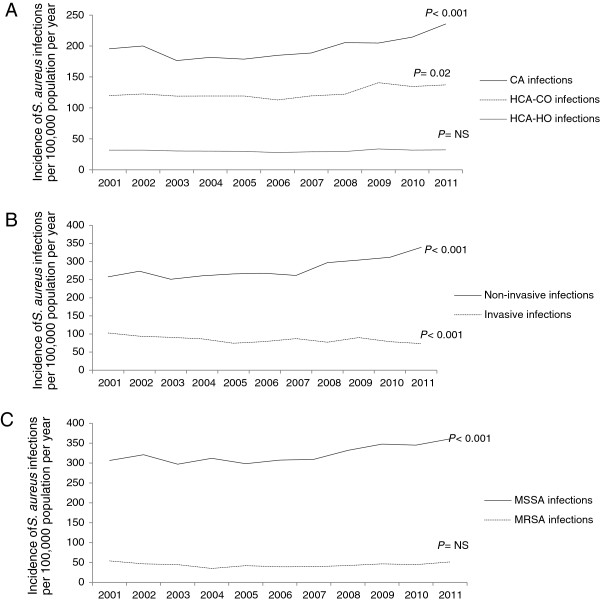
**Incidence of *Staphylococcus aureus *infections per 100,000 population per year in patients hospitalized at Auckland District Health Board, New Zealand, 2001–2011, stratified by (A) place of acquisition; (B) invasive vs. non-invasive infection and (C) MSSA vs. MRSA.** Abbreviations: CA, community-associated; HCA-CO, healthcare-associated, community-onset; HCA-HO, healthcare-associated, hospital-onset; MSSA, methicillin-susceptible *Staphylococcus aureus*; MRSA, methicillin-resistant *Staphylococcus aureus*; NS, not significant.

**Figure 2:**
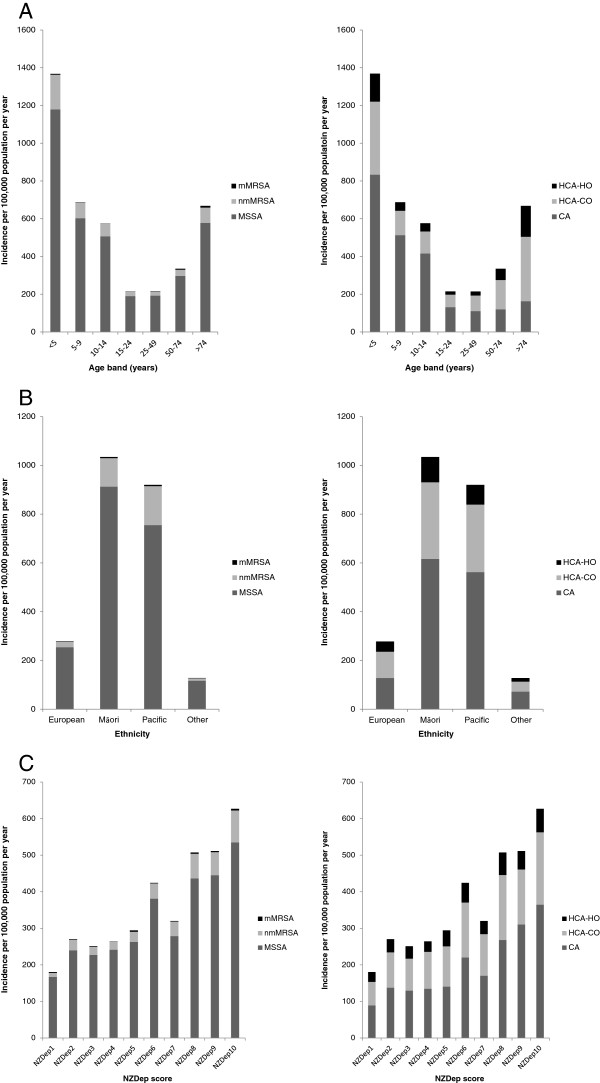
**Incidence of *Staphylococcus aureus *infections per 100,000 population per year in patients hospitalized at Auckland District Health Board, New Zealand, 2001–2011, stratified by (A) age; (B) ethnicity; and (C) NZDep score according to (i) place of acquisition and (ii) MSSA, nmMRSA and mMRSA.** Abbreviations: CA, community-associated; HCA-CO, healthcare-associated, community-onset; HCA-HO, healthcare-associated, hospital-onset; MSSA, methicillin-susceptible *Staphylococcus aureus*; MRSA, methicillin-resistant *Staphylococcus aureus*; NZDep, New Zealand Deprivation Index (1 = least deprived, 10 = most deprived).

Over the 11-year study period there were 26,244 unique *S. aureus* isolates identified from the laboratory database, including 16,249 patients with ≥1 *ICD-10* discharge diagnoses consistent with a *S. aureus*-related clinical syndrome. Of these, 3,752/16,249 (23%) patients were classified as having an invasive infection, and 12,497/16,249 (77%) as having a non-invasive infection. Overall, 8,754/16,249 (54%) of infections were classified as community-associated, 5,523/16,249 (34%) as HCA-CO, and 1,972/16,249 (12%) as HCA-HO (Figure 1). The 11-year averaged incidence of all *S. aureus* related-hospitalizations over the study period was 366 per 100,000 population per year, and increased significantly from 360 per 100,000 population in 2001, to 412 per 100,000 population in 2011 (*P* < 0.001). This rise was related to significant increases in community-associated (*P* < 0.001) and HCA-CO (*P* = 0.02) infections (Figure 1A); non-invasive (*P* < 0.001) infections (Figure 1B); and those caused by MSSA strains (*P* < 0.001) (Figure 1C). The incidence of invasive *S. aureus* infections decreased significantly over the study period from 102 to 73 per 100,000 population per year (*P* < 0.001) (Figure 1B), and there was no significant change in the incidence of HCA-HO infections (Figure 1A). When stratified by age (Figure 2A), the incidence of *S. aureus*-related hospitalizations was highest in the under five and over 75 year age groups (1305 and 646 per 100,000 population per year respectively), and when stratified by ethnicity (Figure 2B), the incidence was highest in Māori and Pacific Peoples (981 and 873 per 100,000 population per year respectively). In addition, when stratified by NZDep score (Figure 2C), the highest incidence of *S. aureus*-related hospitalizations was in those patients residing in areas of high socioeconomic deprivation (higher NZDep scores) (Figure 2C).

### Characteristics of *Staphylococcus aureus* infections in relation to place of acquisition and clinical syndrome

The median age of all patients with *S. aureus* infections was 22 years (interquartile range [IQR] 6–50). There was a statistically significant gradient in the age distribution of patients according to place of acquisition, with the median age highest in patients with HCA-HO infections (median age 40 years, IQR 8–67), and lowest in patients with community-associated infections (median age 13 years, IQR 4–37) (Table 1). The proportion of patients with invasive infections differed significantly according to place of acquisition, with 56% of HCA-HO infections classified as invasive, compared to only 26% of HCA-CO and 14% of community-associated infections (*P* < 0.001). Patients of European ethnicity were more likely to have HCA-HO infections than community-associated infections (54% vs. 36%; *P* < 0.001), whereas Māori or Pacific patients were more likely to have community-associated than HCA-HO infections (22% vs. 17%; *P* < 0.001 and 34% vs. 22%; *P* < 0.001 respectively) (Table 1). In addition, there was variation in the socioeconomic distribution of *S. aureus* infections across acquisition category, such that patients residing in less deprived areas were more likely to have HCA-HO infections than community-associated infections (23% vs. 19%; *P* < 0.001). Conversely, patients in more deprived areas were more likely to have community-associated infections than HCA-HO infections (47% vs. 40%; *P* < 0.001) (Table 1).

**Table 1:** **Characteristics and patient demographics of *Staphylococcus aureus *** **infections, Auckland District Health Board, New Zealand, 2001–2011**

**Characteristic**	**All *S. aureus *** **infections (n = 16,249)**	**Community-onset (CO) (n = 8,754)**	**Healthcare-associated, community-onset (HCA-CO) (n = 5,523)**	**Healthcare-associated, hospital-onset (HCA-HO) (n = 1,972)**	***P *** **value**
Age, median, years (IQR)	22 (6 – 50)	13 (4 – 37)	35 (10 – 60)	40 (8 – 67)	< 0.001^c^
Gender, male	9,098 (56)	4,967 (57)	2,965 (54)	1,166 (59)	< 0.001^d^
Clinical syndrome					
Invasive	3,752 (23)	1,220 (14)	1,427 (26)	1,105 (56)	< 0.001^d^
Non-invasive	12,497 (77)	7,534 (86)	4,096 (74)	867 (44)	-
Ethnicity^a^					
European	6,824 (42)	3,114 (36)	2,658 (48)	1,052 (54)	< 0.001^d^
Māori	3,211 (20)	1,908 (22)	978 (18)	325 (17)	< 0.001^d^
Pacific peoples	4,802 (30)	2,932 (34)	1,444 (26)	426 (22)	< 0.001^d^
Other	1,327 (8)	743 (8)	427 (8)	157 (8)	0.25^d^
NZDep^b^					
1–3	3,093 (20)	1,553 (19)	1,105 (21)	435 (23)	< 0.001 ^d^
4–7	5,443 (35)	2,780 (34)	1,976 (37)	687 (37)	
8–10	6,861 (45)	3,915 (47)	2,202 (42)	744 (40)	0.015 ^d^
					< 0.001 ^d^

^a^Data available for 16,164 infections (8,697 CO; 5,507 HCA-CO; 1,960 HCA-HO).

^b^Data available for 15,397 infections (8,248 CO; 5,283 HCA-CO; 1,866 HCA-HO).

^c^*P* value calculated using Kruskal-Wallis test.

^d^*P* value calculated using χ^2^ test.

Abbreviations: *IQR* interquartile range, *MSSA* methicillin-susceptible *Staphylococcus aureus*, *MRSA* methicillin-resistant *Staphylococcus aureus*, *CO* community-onset, *HCA-CO* healthcare-associated, community-onset, *HCA-HO* healthcare-associated, hospital-onset.

### Comparison of MSSA, nmMRSA and mMRSA infections

The incidence of MSSA infections increased significantly from 306 per 100,000 in 2001 to 361 per 100,000 in 2011 (*P* < 0.001). In contrast, the incidence of MRSA did not increase over the study period (Figure 1). Overall, 1,957/16,249 (12%) of infections were caused by MRSA (1,804 [94%] nmMRSA and 117 [6%] mMRSA). There were distinct demographic differences between patients with MSSA, nmMRSA and mMRSA infections, such that patients with mMRSA infections were significantly older than patients with either MSSA (median 52 years vs. 22 years; *P* < 0.001) or nmMRSA (median 52 years vs. 16 years; *P* < 0.001), and were more likely to have invasive infections than patients with MSSA (44% vs. 24%; *P* < 0.001) or nmMRSA (44% vs. 17%; *P* < 0.001). Patients with MSSA or nmMRSA did not differ significantly according to the place of acquisition of their infection (Table 2). However, when compared to patients with nmMRSA, patients with mMRSA infections were significantly more likely to have HCA-CO or HCA-HO infections (50% vs. 33%; *P* < 0.001 and 25% vs. 13%; *P* < 0.001, respectively).

**Table 2:** **Patient characteristics associated with methicillin-susceptible *Staphylococcus aureus *** **(MSSA) infections, non-multidrug resistant methicillin resistant *S. aureus *** **(nmMRSA) infections and multidrug resistant MRSA (mMRSA) infections, Auckland District Health Board, New Zealand, 2001-2011**

**Characteristic**	**MSSA (n = 14,292)**	**nmMRSA (n = 1840)**	**mMRSA (n = 117)**	***P***	**OR of MSSA vs. nmMRSA (95% CI)**	***P***	**OR nmMRSA vs. mMRSA infection (95% CI)**
Age, median, IQR^a^	22 (6–50)	16 (3–47)	52 (31.5–71.5)	< 0.001	-	< 0.001	-
Male sex^b^	8,028 (56)	1,005 (55)	69 (59)	0.34	1.19 (0.83–1.69)	0.23	0.85–1.04
Clinical syndrome^b^							
Invasive	3,390 (24)	311 (17)	51 (44)	< 0.001	1.53 (1.35–1.74)	< 0.001	0.26 (0.18–0.39)
Non-invasive	10,902 (76)	1,529 (83)	66 (56)	-	-	-	-
Place of acquisition^b^							
CO	7,732 (54)	993 (54)	29 (25)	0.93	1.0 (0.91–1.11)	< 0.001	3.56 (2.32–5.47)
HCA-CO	4,851 (34)	614 (33)	58 (50)	0.64	1.03 (0.93–1.14)	< 0.001	0.51 (0.35–0.74)
HCA-HO	1,709 (12)	233 (13)	30 (25)	0.40	0.94 (0.81–1.08)	< 0.001	0.42 (0.27–0.65)
Ethnicity^b, c^							
European	6,223 (44)	544 (30)	57 (49)	< 0.001	1.84 (1.65–2.04)	< 0.001	0.44 (0.30–0.64)
Māori	2,845 (20)	349 (19)	17 (15)	0.37	1.06 (0.94–1.19)	0.27	1.37 (0.81–2.33)
Pacific Peoples	3,942 (28)	832 (46)	28 (24)	< 0.001	0.41 (0.37–0.46)	< 0.001	2.92 (1.89–4.51)
Other	1,210 (8)	103 (5)	14 (12)	< 0.001	1.56 (1.27–1.92)	0.013	0.44 (0.24–0.79)
NZDep band^b,d^							
1–3	2,792 (21)	275 (16)	26 (25)	< 0.001	1.37 (1.20-1.57)	0.029	0.58 (0.37-0.92)
4–7	4,864 (36)	546 (32)	33 (31)	< 0.001	1.21 (1.09-1.35)	1.0	1.02 (0.67-1.56)
8–10	5,903 (44)	911 (53)	47 (44)	< 0.001	0.69 (0.63-0.77)	0.11	1.39 (0.94-2.07)

^a^*P* value calculated using Kruskal Wallis test.

^b^*P* value calculated using χ^2^ test.

^c^Data available for 16,164 infections (14,220 MSSA, 1,828 nmMRSA and 116 mMRSA).

^d^Data available for 15,397 infections (13,559 MSSA, 1,732 nmMRSA and 106 mMRSA).

Abbreviations: *OR* odds ratio, *CI* confidence interval, *IQR* interquartile range, *MSSA* methicillin-susceptible *Staphylococcus aureus*, *nmMRSA* non-multidrug resistant methicillin-resistant *Staphylococcus aureus*, *mMRSA* multidrug resistant methicillin-resistant *Staphylococcus aureus*, *CO* community-onset, *HCA-CO* healthcare-associated, community-onset, *HCA-HO* healthcare-associated, hospital-onset.

Given that the number of patients with mMRSA infections was relatively small (n = 117) compared to patients with MSSA (n = 14,292) or nmMRSA (n = 1,840), we concentrated on comparing risk factors associated with either MSSA or nmMRSA infections (Table 3). On multivariate analysis, factors associated with nmMRSA isolation were: age over 75 years (odds ratio (OR), 1.77 [95% CI, 1.44-2.17]); Māori or Pacific ethnicity (OR, 1.48 [95% CI, 1.26-1.75] and OR, 2.41 [95% CI, 2.09-2.78] respectively); and HCA-HO infection (OR, 1.37 [95% CI, 1.15-1.62]). In addition, nmMRSA infections were less likely to be invasive than MSSA infections (OR 0.64 [95% CI, 0.55-0.74]).

**Table 3:** **Multivariate analysis of factors associated with non-multidrug resistant methicillin-resistant *Staphylococcus aureus *** **(nmMRSA) infections, Auckland District Health Board, New Zealand, 2001-2011**

**Characteristic**	**OR of nmMRSA vs. MSSA infection (95% CI)**
Sex	
Male	Reference
Female	0.96 (0.86–1.06)
Type of infection	
Non-invasive	Reference
Invasive	0.64 (0.55–0.74)
Age band (years)	
15 to 49	Reference
Under 5	0.98 (0.85–1.14)
5 to 14	1.12 (0.97–1.29)
50 to 74	1.09 (0.92–1.08)
Over 75	1.77 (1.44–2.17)
Ethnicity	
European	Reference
Māori	1.48 (1.26–1.75)
Pacific peoples	2.41 (2.09–2.78)
Other	0.99 (0.79–1.25)
NZDep score	
1–3 (low)	Reference
4–7 (medium)	0.98 (0.84–1.15)
8–10 (high)	1.10 (1.14–1.29)
Place of acquisition	
CO	Reference
HCA-CO	1.08 (0.96–1.21)
HCA-HO	1.37 (1.15–1.62)

Abbreviations: *OR* odds ratio, *CI* confidence interval, *MSSA* methicillin-susceptible *Staphylococcus aureus*, *nmMRSA* non-multidrug resistant methicillin-resistant *Staphylococcus aureus*, *CO* community-onset, *HCA-CO* healthcare-associated, community-onset, *HCA-HO* healthcare-associated, hospital-onset.

## Discussion

In this study, we systematically assessed the trends, incidence and demographics of patients hospitalized with *S. aureus* infections in our setting. We found notable demographic differences between community and healthcare-associated *S. aureus* infections, as well as differences in the comparative epidemiology of MSSA and MRSA. Our findings have implications for the following reasons.

First, by assessing all *S. aureus* infections, rather than just one specific aspect such as MRSA or nosocomial infections, our data provide a broad and comprehensive representation of the trends and burden of *S. aureus* disease in our setting. We observed an increase in the overall incidence of *S. aureus* infection, largely driven by an increase in community-associated non-invasive infections. Our finding of a significant increase in the incidence of hospitalizations for skin infections is in keeping with other recent studies in New Zealand [11,23,24], and further highlights this concerning national trend. Although reasons for this increase are unclear, suggested contributory factors include delayed access to healthcare, increases in household crowding and declining socioeconomic circumstances for specific population groups [23,24]. However, in contrast to the increase in non-invasive infections, the incidence of invasive *S. aureus* infections decreased significantly over the study period from 102 to 73 per 100,000 population per year (*P* < 0.001). This trend is in keeping with recent reports from North America, [10,27,28] and may, in part, be due to improved local infection control practices, such as improvements in hand hygiene compliance and measures to reduce healthcare-associated bloodstream infections, including those caused by *S. aureus*[29].

Second, we found that in our setting, the vast majority (88%) of *S. aureus* infections were caused by MSSA, and the incidence of MSSA increased significantly over the study period. Conversely, the incidence of MRSA infections remained stable; therefore the percentage contribution of MRSA to the overall burden of *S. aureus* disease actually decreased. This finding is in sharp contrast to North America, where studies suggest approximately 40-60% of *S. aureus* infections are caused by MRSA, largely due to the epidemic spread of the USA300 clone [10,28]. However, despite the large burden of MSSA infections in our setting, relatively little is known about the molecular epidemiology of MSSA strains in New Zealand. In particular, the extent to which the observed increase in community-associated MSSA SSTI is driven by the spread of one MSSA clone, or is due to a range of MSSA lineages is unknown. Interestingly, after multivariate analysis, we found that nmMRSA infections were more common than MSSA infections in Māori and Pacific Peoples compared to European patients. The reasons for this observation are unclear, although numerous studies have described an association between nmMRSA isolation and other distinct ethnic groups, for example African-Americans [28] and Australian Aboriginals [21]. Moreover, an association between specific community-associated MRSA clones and Indigenous ethnic groups has recently been demonstrated in New Zealand [17]. Future work should investigate the possible host, bacterial and socioenvironmental factors that predispose such groups to infection with certain MRSA clones.

In our locale, patients with multiresistant MRSA represented a distinct epidemiological group, in that they were older, more likely to be European and more likely to have healthcare-associated infections than patients with MSSA or nmMRSA. This finding is consistent with a recent study from our setting, which showed that specific multiresistant MRSA clones (e.g. sequence type (ST) 22 MRSA and ST239 MRSA) were associated with prior healthcare exposure, and were more likely to be isolated from older European patients [17]. It is possible that our observation may reflect circulation and acquisition of mMRSA in long-term residential care facilities, with elderly European patients markedly overrepresented in long-term residential care facilities in New Zealand compared to non-European and Indigenous groups [30].

There were several limitations with our study. In particular, our analysis was restricted to patients with *S. aureus*-related hospitalizations, and did not include patients who only received treatment in primary care. Furthermore, we used a relatively conservative and specific method of case ascertainment in that we only included patients who had culture-proven *S. aureus* disease plus a hospital discharge code describing a *S. aureus*-related clinical syndrome, rather than all patients who had a positive *S. aureus* culture. Moreover, most patients discharged from hospital with an infectious disease diagnosis do not have any identified etiological agent [24]. As such, the true burden of *S. aureus* disease in our setting is likely to be considerably higher. In addition, we did not have patient-level information on factors such as medical co-morbidities or antibiotic usage. However, our main aim was not to provide detailed descriptive information on specific clinical syndromes, but rather to provide a broad overview of the demographics and trends of a large number of *S. aureus* infections.

## Conclusions

In conclusion, our study provides valuable baseline information on the epidemiology and trends of *S. aureus* infections in a New Zealand population. There were notable sociodemographic differences in disease burden, with the incidence highest in Māori and Pacific Peoples. Importantly, we observed a steady and significant increase in *S. aureus* infections, predominantly due to a rise in non-invasive community-associated MSSA. Future work should investigate the clinical and molecular epidemiological associations underlying this concerning trend.

## Competing interests

The authors declare that they have no competing interests.

## Authors’ contributions

DAW conceived the study, participated in data collection and analysis, and drafted the manuscript. AL participated in data collection and analysis. MGT, MGB, SAR and SRR provided intellectual contributions to the manuscript sufficient to justify authorship. All authors read and approved the final manuscript.

## Pre-publication history

The pre-publication history for this paper can be accessed here:

http://www.biomedcentral.com/1471-2334/13/569/prepub

## Supplementary Material

Additional file 1 **International Classification of Diseases, Tenth Revision *(ICD-10) *** **codes for *Staphylococcus aureus*–related infections and associated clinical syndromes.** Click here for file
