# Supplementary material for: Incidence, trends and demographics of Staphylococcus aureus infections in Auckland, New Zealand, 2001–2011
Source: BMC Infect Dis. 2013 Dec 3;13:569. doi: 10.1186/1471-2334-13-569 (PMC4219404; doi:10.1186/1471-2334-13-569)
Supplement: Additional file 1 — International Classification of Diseases, Tenth Revision (ICD-10) codes for Staphylococcus aureus –related infections and associated clinical syndromes. [file 1471-2334-13-569-S1.docx]

**Additional file 1. International Classification of Diseases, Tenth Revision *(ICD-10-CM)* codes for *Staphylococcus aureus*–related infections and associated clinical syndromes**

| ***ICD-10-CM* codes and associated clinical syndromes** |
| --- |
| **Skin and soft tissue infection**  L01.0, L01.1 – Impetigo  L02.0-L02.9 – Cutaneous abscess, furuncle and carbuncle  L03.01-L03.9 – Cellulitis  L0.40-L04.9 – Acute lymphadenitis  L05.0 – Pilonidal cyst with abscess  L08.0 – Pyoderma  L08.1, L08.8, L08.9 – Other infections of skin and subcutaneous tissue  L30.3, 30.8, L30.9 – Dermatitis unspecified and other specified (eczema) and infective eczema |
| **Musculoskeletal infection**  M00.0 – Staphylococcal arthritis and polyarthritis  M00.9 – Pyogenic arthritis, unspecified  M46.2 – Osteomyelitis of vertebra  M46.3 – Infection of intervertebral disc  M46.5 – Other infective spondylopathy  M60.0 – Infective myositis  M60.8 – Other myositis  M60.9 – Myositis, unspecified  M70.1-M70.7 – Bursitis  M71.0-M71.1 – Abscess of bursa, other infective bursitis  M86.0-M86.9 – Osteomyelitis |
| **Respiratory infection**  J15.2 – Pneumonia due to staphylococcus  J15.9 – Bacterial pneumonia, unspecified  J86.0 – Pyothorax with fistula  J86.9 – Pyothorax without fistula  J85.0 – Gangrene and necrosis of lung  J85.1 – Abscess of lung with pneumonia  J85.2 – Abscess of lung without pneumonia  P23.2 – Congenital pneumonia due to staphylococcus |
| **Endovascular infection**  I33.0 – Acute and subacute infective endocarditis  I33.9 – Acute endocarditis, unspecified  I38 – Endocarditis, valve unspecified  I39.8 – Endocarditis, valve unspecified, in diseases classified elsewhere  T82.6-T82.9 – Infection and inflammatory reaction due to cardiac valve prosthesis |
| **Central nervous system infection**  G00.3 – Staphylococcal meningitis  G06.0-G06.2 – Intracranial and intraspinal abscess and granuloma |
| **Sepsis / bacteremia ^a^**  A41.0 – Sepsis due to *Staphylococcus aureus*  P36.2 – Sepsis of newborn due to *Staphylococcus aureus* |

^a^ Case definition also included all *Staphylococcus aureus* bloodstream isolates.
